# Feeding tube use is associated with severe scoliosis in patients with cerebral palsy and limited ambulatory ability

**DOI:** 10.1007/s43390-022-00540-6

**Published:** 2022-06-28

**Authors:** Nicholas Yoo, Brian Arand, Junxin Shi, Jingzhen Yang, Garey Noritz, Amanda T. Whitaker

**Affiliations:** 1 College of Medicine, The Ohio State University, Columbus, OH USA; 2 Nationwide Children’s Hospital, Columbus, OH USA; 3 Shriners Hospital Northern California, University of California Davis, Sacramento, CA USA

**Keywords:** Neuromuscular scoliosis, Cerebral palsy, Feeding tube, Scoliosis progression

## Abstract

**Purpose:**

Cerebral palsy (CP) is the most common motor disorder in childhood. Scoliosis is a common complication of CP that can reach clinically severe levels, but predictors for scoliosis in CP are not well understood. Some variables identified in the literature involve the severity of the brain injury and the presence of hip deformity. We aimed to identify associations with developing severe scoliosis in a prospective cohort of patients with cerebral palsy at higher risk for severe curve progression.

**Methods:**

This study reviewed a prospectively collected database at a tertiary children’s hospital. We evaluated a panel of potential associations with severe scoliosis—including age, sex, Gross Motor Function Classification System (GMFCS) class, history of hip surgery, epilepsy, and feeding tube presence—in a population of children with limited ambulatory ability defined as GMFCS level IV or V CP. Univariate analysis and multivariate logistic regression with stepwise selection was used for analysis.

**Results:**

Descriptive analysis showed that female sex, higher GMFCS class, history of hip surgery, non-upright seating, pelvic obliquity, presence of epilepsy, and presence of a feeding tube were associated with an increased risk for scoliosis. Multivariate logistic regression analysis revealed that the presence of a feeding tube was associated with severe scoliosis even when controlling for GMFCS and age.

**Conclusions:**

Feeding tube use may stratify risk for severe scoliosis progression in patients with GMFCS IV or V CP.

## Introduction

Cerebral palsy (CP) is a group of permanent, non-progressive, activity-limiting disorders of movement and posture that are attributed to disturbances to the developing fetal or infant brain [1]. Occurring in about 2 per 1000 live births, CP is the most common motor disorder of childhood [2]. Scoliosis is a common complication in CP, present in about 25% of patients [3, 4]. Scoliosis in CP can affect the skin, gastrointestinal and cardiopulmonary systems leading to significant morbidity [5–7]. Natural history studies have demonstrated that children who reach higher Cobb angles before adulthood are at higher risk of progression, which can reach as high as 130 degrees [8–11]. A systematic review published in 2010 noted a paucity of studies above Sackett level IV on risk factors for severe scoliosis and variable methodology between existing studies [12].

Scoliosis risk correlates with global disease severity. The significant association for severe scoliosis in patients with CP is the severity of the motor limitations as classified by the Gross Motor Function Classification System (GMFCS). This commonly used system categorizes motor function by age-appropriate activities. Patients classified at Level I are the most ambulatory, while patients classified at Level IV and V use wheelchairs as their primary means of mobility [13]. Epidemiological data from the Swedish Cerebral Palsy registry have shown that patients classified at GMFCS levels III, IV or V have a markedly increased risk for scoliosis relative to patients classified at levels I or II [3, 4]. However, among patients within the higher risk GMFCS classes, no additional risk stratification exists. Other reported associations with scoliosis in all patients with CP include female sex [3, 14–16] epilepsy [14–16], and history of hip surgery [15, 16].

Proposed mechanisms for scoliosis in patients with CP are tonal asymmetry [5, 17] and trunk imbalance [18], suggested by the co-occurrence of windswept hips (defined as one hip abducted and externally rotated while the other is adducted and internally rotated), pelvic obliquity, and scoliosis [17, 19–21]. Asymmetric, limited hip flexion [22] and limited knee extension [14] have both been associated with scoliosis in CP. Hip surveillance programs may be associated with a decrease in scoliosis incidence [3, 17, 20], although there are no published spine surveillance programs.

Custom seating arrangements and bracing may help maintain an upright position during childhood [6], but the only definitive treatment for scoliosis in CP is surgery [23]. Surgical indications include a severe curve and functional seating impairment, although there are no consensus criteria, and complication rates for scoliosis surgery in CP are significant [23]. The medical benefits of scoliosis surgery are improved sitting balance, less wheelchair modifications, and improved weight gain in children with CP below the 50 percentile for BMI on CP-specific growth charts [23, 24]. Surgery may also be associated with improved caregiver quality-of-life; however, patient outcomes are often difficult to assess because of limited communication ability in many patients [23, 25, 26].

No spine surveillance guidelines exist in CP as they do for hip surveillance [23, 27]. More robust risk stratification for scoliosis progression is needed in higher risk populations—i.e., patients at higher GMFCS classes—to aid families and clinicians in creating appropriate surveillance schedules, as there are potential harms of over-monitoring, such as radiation exposure, and under-monitoring, such as delayed surgical intervention and increased risk. This study aims to evaluate elements of clinical history, physical exam, and imaging as potential associations for severe scoliosis progression in patients with GMFCS IV/V CP.

## Methods

### Patients

This study reviewed and extracted the data from a prospectively collected database of the Cerebral Palsy Research Network (CPRN) [28]. Our study population included all patients seen at a single tertiary children’s hospital with GMFCS IV or V cerebral palsy who were between 5 and 18 years old. This work was approved by the Institutional Review Board at Nationwide Children’s Hospital.

### Data collection

This study analyzed the following potential predictors for severe scoliosis: GMFCS level, age, sex, hip migration greater than 30%, history of hip surgery, history of selective dorsal rhizotomy, pelvic obliquity, presence of epilepsy, presence of a feeding tube, upright seating, Galeazzi sign, hip extension range of motion (ROM) symmetry, hip flexion ROM asymmetry, hip abduction ROM asymmetry, hip extension spasticity asymmetry, hip flexion spasticity asymmetry, hip abduction spasticity asymmetry, as well as ROM and spasticity symmetry for internal rotation, external rotation, and adduction.

Range of motion measurements within five-degree intervals for both hips were considered symmetric; otherwise, they were considered asymmetric. Spasticity was assessed using the Ashworth scale [29]. Equal Ashworth scores for both hips were classified as symmetric; otherwise, patients were classified as having asymmetric spasticity. The presence of Galeazzi sign, epilepsy, pelvic obliquity, and feeding tube (specifically, gastrostomy and gastrojejunostomy tubes) were recorded as binary variables. Seating was a binary variable, where “seated upright” was considered normal while “leans left,” “leans right,” and “leans forward” were considered abnormal.

Hip surgeries, both bony and soft tissue, and selective dorsal rhizotomies were abstracted from orthopedic and neurosurgical operative notes and progress notes. Hip migration percentages were either abstracted from orthopedic progress notes or retrospectively measured using the HipScreen app [30]. Migration percentage greater than 30% in either hip was considered positive.

Our outcome variable was a Cobb angle of greater than or equal to 40°. This cutoff was chosen based on natural history studies that demonstrated that children who reach Cobb angles of 30–50° before adulthood are at higher risk of further progression [8–11]. We specifically chose 40° to maintain consistency with the previous studies that correlated GMFCS class with scoliosis risk [3, 4]. Cobb angles from PA-scoliosis films were abstracted from orthopedic progress notes or radiology reports. For patients without dedicated PA-scoliosis films, Cobb angles from other films (e.g., abdominal X-rays or chest X-rays) were either retrospectively abstracted or measured by the lead author (NY). Negative clinical exams for scoliosis were included as “non-severe” scoliosis [3].

### Design and statistics

Descriptive analysis was conducted to describe the demographic characteristics of the patients, and the distributions of the outcome variable, severe scoliosis (i.e., presence of Cobb angle greater 40°), by demographic and clinically relevant variables. *P* values were calculated using either chi-squared tests or Fisher’s exact tests for categorical variables and independent *t* tests for continuous variables. We built our multivariate logistic regression model based on the results from previously published studies [3, 14–16], the clinical experience of the authors, and *p* values from the univariate analysis. We included the following variables in our multivariate logistic regression model: GMFCS, sex, hip migration > 30%, hip surgery, selective dorsal rhizotomy, epilepsy presence, and feeding tube presence. We also included a continuous variable of age at the end of follow-up in this model. To identify the most important predictors of severe scoliosis, we further analyzed the multivariate logistic regression model using stepwise selection, where the significance levels used for a variable to be entered and stayed in the model were both 0.15. We also calculated the area under the ROC curve for both multivariate logistic regression models. All statistical analyses were performed using SAS (Statistical Analysis Software 9.4, SAS Institute Inc, Cary, North Carolina, USA). The level of statistical significance was set at *p* < 0.05.

## Results

There were 1157 patients in the database, and 277 met the inclusion criteria. We excluded nine patients with no scoliosis assessments, bringing the final total to 268. There was an equal number of patients (134, 50%) at GMFCS IV and GMFCS V (Table 1). The study group had 163 (61%) males and 105 (39%) females, and the average age was 10.9 ± 3.7, with no significant difference in age between patients with and without severe scoliosis (Table 1). Fifty-two patients (19%) developed severe scoliosis (Table 1).

**Table 1:** Severe scoliosis by demographic and clinical variables

	Total (*n* = 268)	Severe scoliosis (*n* = 52)	No severe scoliosis (*n* = 216)	*p* value
	*N* (%)	*N* (%)	*N* (%)	
Age (mean ± SD)	10.9 ± 3.7	11.3 ± 3.2	10.8 ± 3.8	0.33
Sex				0.036*
F	105 (39)	27 (52)	78 (36)	
M	163 (61)	25 (48)	138 (64)	
GMFCS				< 0.0001*
IV	134 (50)	10 (19)	124 (57)	
V	134 (50)	42 (81)	92 (43)	
Feeding tube present				0.0001*
No	103 (38)	8 (15)	95 (44)	
Yes	125 (47)	35 (67)	90 (42)	
Missing	40 (15)	9 (17)	31 (14)	
Epilepsy present				0.043*
No	57 (21)	6 (12)	51 (24)	
Yes	129 (48)	30 (58)	99 (46)	
Missing	82 (31)	16 (31)	66 (31)	
Pelvic obliquity				0.0033* **
No	76 (28)	7 (13)	69 (32)	
Yes	17 (6)	7 (13)	10 (5)	
Missing	175 (65)	38 (73)	137 (63)	
Upright seating				0.016*
No	33 (12)	8 (15)	25 (12)	
Yes	69 (26)	5 (10)	64 (30)	
Missing	166 (62)	39 (75)	127 (59)	
Hip surgery				0.001*
No	162 (60)	21 (40)	141 (65)	
Yes	106 (40)	31 (60)	75 (35)	
Hip migration > 30%				0.45
No	126 (47)	22 (42)	104 (48)	
Yes	142 (53)	30 (58)	112 (52)	
Selective Dorsal Rhizotomy				0.32**
No	254 (95)	51 (98)	203 (94)	
Yes	14 (5)	1 (2)	13 (6)	
Extension ROM Sym				0.31
No	33 (12)	8 (15)	25 (12)	
Yes	172 (64)	29 (56)	143 (66)	
Missing	63 (24)	15 (29)	48 (22)	
Flexion ROM Sym				0.22**
No	18 (7)	6 (12)	12 (6)	
Yes	179 (67)	35 (67)	144 (67)	
Missing	71 (26)	11 (21)	60 (28)	
Abduction ROM Sym				0.082
No	32 (12)	10 (19)	22 (10)	
Yes	121 (45)	21 (40)	100 (46)	
Missing	115 (43)	21 (40)	94 (44)	
Extension Ashworth Sym				0.18**
No	8 (3)	3 (6)	5 (2)	
Yes	217 (81)	40 (77)	177 (82)	
Missing	43 (16)	9 (17)	34 (16)	
Flexion Ashworth Sym				1.0**
No	5 (2)	1 (2)	4 (2)	
Yes	220 (82)	42 (81)	178 (82)	
Missing	43 (16)	9 (17)	34 (16)	
Abduction Ashworth Sym				0.35**
No	18 (7)	5 (10)	13 (6)	
Yes	207 (77)	38 (73)	169 (78)	
Missing	43 (16)	9 (17)	34 (16)	

Frequency (*N*), proportion (%), and *p* values of predictors in all patients and patients with and without severe scoliosis. *Denotes significant values (*p* < 0.05) ** Denotes *p* values calculated by Fisher’s exact test. *P* value for age was calculated by independent *t* test. All other *p* values calculated by chi-squared tests

On descriptive analysis, males had a lower risk than females (*p* = 0.036) for severe scoliosis (Table 1). Patients with GMFCS V had significantly greater risk than patients with GMFCS IV (*p* < 0.0001). Epilepsy (*p* = 0.043), pelvic obliquity (*p* = 0.0033), presence of a feeding tube (*p* = 0.0001), and history of hip surgery (*p* = 0.0010) were associated with greater odds of severe scoliosis than their respective counterparts, while upright seating was associated with lower odds of severe scoliosis (*p* = 0.016) than abnormal seating. (Table 1). All other variables were not associated with an increased risk of severe scoliosis.

Multivariate logistic regression of GMFCS, age, sex, hip migration, hip surgery, selective dorsal rhizotomy, epilepsy, and feeding tube presence showed that only GMFCS (OR 4.0, 95% CI 1.4–11.0) and age (OR 1.2, 95% CI 1.0–1.3) were associated with greater risk of severe scoliosis (Table 2). The area under the curve for this model was 0.81 (Table 2).

**Table 2:** Multivariate logistic regression model with select variables

	OR	95% CI
GMFCS	4.0	1.4–11.0
Age	1.2	1.0–1.3
Sex	1.0	0.4–2.3
Hip migration	0.91	0.4–2.1
Hip surgery	2.2	0.9–5.3
Selective Dorsal Rhizotomy	< 0.001	< 0.001– > 999
Epilepsy present	1.7	0.6–4.8
Feeding tube present	2.4	0.9–6.9

Point estimate for odds ratio (OR) and 95% Wald confidence intervals (CI) for the model

Logistic regression with stepwise selection showed that age (OR 1.2, 95% CI 1.1–1.4), GMFCS (OR 4.9, 95% CI 1.9–12.8), and presence of a feeding tube (OR 3.0, 95% CI 1.1–8.0) were the only predictors remaining, all of which were statistically significant (Table 3). The area under the curve for this model was 0.78.

**Table 3:** Multivariable logistic regression model after stepwise selection

	OR	95% CI
GMFCS	4.9	1.9–12.8
Age	1.2	1.1–1.4
Feeding tube present	3.0	1.1–8.0

Point estimate for odds ratio (OR) and 95% Wald confidence intervals (CI) for the model

## Discussion

The goal of this study was to identify variables that stratify severe scoliosis rates among patients with GMFCS IV and V cerebral palsy. Based on the literature, we chose study variables that correlated with disease severity and variables related to hip abnormalities. Demographically, the population we studied was similar to that of Hagglund et al. [3].

The descriptive analysis found one previously unreported association with severe scoliosis: the presence of a feeding tube. Consistent with several recent studies, we found epilepsy was associated with severe scoliosis, [14–16]. We also found that a history of hip surgery was associated with progression to severe scoliosis, which had been reported in some studies [15, 16] but not in others [14]. Our finding that pelvic obliquity is associated with scoliosis is consistent with previous reports [17, 19–21].

Multivariate analysis with stepwise selection did not support epilepsy, female sex, or hip surgery as independent associations. Epilepsy, female sex, and hip surgery had been reported in studies that included patients at all GMFCS levels, but GMFCS classes IV or V were associated with relatively greater risk than epilepsy and female sex in those studies [3, 14–16]. Thus, these results may aid in the development of a spine-monitoring program that is specific to patients in higher GMFCS classes.

We hoped the data on range of motion and spasticity could build on previous studies that suggested a clinically relevant relationship between hip, pelvis, and spine deformity [15–17, 20, 22]. However, we did not find that these variables were associated with severe scoliosis. The presence of hip deformity may not be an association in this population—GMFCS and feeding tube use together suggest that rates of severe scoliosis may correlate with the severity of brain injury.

The presence of a feeding tube as an independent association with severe scoliosis was a novel finding. This finding was consistent with Bertoncelli et al. showing that severe neuromuscular scoliosis may be associated with children who required a gastrostomy tube placement in adolescents [31]. Feeding status and scoliosis severity might be generally linked: scoliosis surgery has been shown to correct low BMI in CP [24], and the presence of a g-tube is associated with an increased risk of life-threatening complications (i.e., Clavien–Dindo Grade IV) following scoliosis surgery [32]. This novel association may aid risk stratification for scoliosis in patients at higher risk GMFCS classes.

We acknowledged several limitations. As demonstrated by relatively wide confidence intervals, this study was likely to be underpowered, given the large number of variables being assessed. In addition, the sample for this study was drawn from a specialty care clinic at a single tertiary children’s hospital. The results of this study may not be generalizable to the overall population of children with GMFCS IV/V CP.

G-tube use is a novel correlate for severe scoliosis in a population of patients with CP already at higher risk. Further research is required to definitively establish predictors for progression to severe scoliosis in CP, predictors which could aid clinicians and families in planning care and could establish scoliosis screening guidelines.

## Data Availability

All data are available for review upon request.
